# An Update on Plant Photobiology and Implications for Cannabis Production

**DOI:** 10.3389/fpls.2019.00296

**Published:** 2019-03-29

**Authors:** Samuel Eichhorn Bilodeau, Bo-Sen Wu, Anne-Sophie Rufyikiri, Sarah MacPherson, Mark Lefsrud

**Affiliations:** Department of Bioresource Engineering, McGill University, Montreal, QC, Canada

**Keywords:** cannabis, *Cannabis sativa*, HPS, LEDs, light, photobiology, photomorphology, photosynthesis

## Abstract

This review presents recent developments in plant photobiology and lighting systems for horticultural crops, as well as potential applications for cannabis (*Cannabis sativa* and *C. indica*) plant production. The legal and commercial production of the cannabis plant is a relatively new, rapidly growing, and highly profitable industry in Europe and North America. However, more knowledge transfer from plant studies and horticultural communities to commercial cannabis plant growers is needed. Plant photosynthesis and photomorphogenesis are influenced by light wavelength, intensity, and photoperiod *via* plant photoreceptors that sense light and control plant growth. Further, light properties play a critical role in plant vegetative growth and reproductive (flowering) developmental stages, as well as in biomass, secondary metabolite synthesis, and accumulation. Advantages and disadvantages of widespread greenhouse lighting systems that use high pressure sodium lamps or light emitting diode (LED) lighting are known. Some artificial plant lighting practices will require improvements for cannabis production. By manipulating LED light spectra and stimulating specific plant photoreceptors, it may be possible to minimize operation costs while maximizing cannabis biomass and cannabinoid yield, including tetrahydrocannabinol (or Δ^9^-tetrahydrocannabinol) and cannabidiol for medicinal and recreational purposes. The basics of plant photobiology (photosynthesis and photomorphogenesis) and electrical lighting systems are discussed, with an emphasis on how the light spectrum and lighting strategies could influence cannabis production and secondary compound accumulation.

## Introduction

The legal status of cannabis production is shifting, causing a rapidly expanding market in both North America and Europe. Canada has become the second country in the world to legalize the use of both medicinal and recreational cannabis (Dyer, 2018). Such full legalization allows industry and researchers to work together to explore the uncharted science of this once-forbidden plant. Although cannabis (*Cannabis sativa ssp.*) has been harvested for food (seeds), fiber (stems), and medicine (buds) throughout most of human history (Mercuri et al., 2002; Clarke and Merlin, 2013), its listing as an illegal drug to date has left little published scientific literature.

Commercial cannabis production typically occurs indoors and requires environmental controls such as humidity and lighting for both vegetative growth and budding (flowering) developmental stages (Hillig, 2005). During the vegetative growth stage, high light intensity is needed to maximize cannabis growth and proper photoperiodicity control is necessary to initiate budding (Arnold, 2013). Growing cannabis plants solely with indoor lighting allows a continuous and uniform cannabinoid yield for high-quality products, but it requires high-energy inputs. As such, indoor cannabis production has been classified as one of the most energy-intensive industries in the US (Warren, 2015). In this regard, the selection of electrical lighting systems and light spectra are of utmost importance, as they determine operation costs and consequent product pricing.

In the general horticultural industry, growers use different light spectra and intensities to influence plant morphology, secondary metabolism, and flowering (Lefsrud et al., 2008; Kohyama et al., 2014; Wang et al., 2016). However, commercial growers in the cannabis industry are still referring to unreliable information, given the lack of peer-reviewed reports on cannabis production. Exceptionally, it has been reported that reducing the photoperiod to approximately 12 h is a common practice in the cannabis production industry to initiate flowering (Chandra et al., 2017). For other commonly grown flowering plants in the horticultural industry, flowering is initiated *via* night interruption (Yamada et al., 2008; Blanchard and Runkle, 2010; Park et al., 2016). Both methods initiate flowering; however, reducing photoperiod potentially leads to plant yield reduction.

With decades of research committed to understanding the impact of narrow light spectra on plant growth, the basis of wavelength effect on photosynthesis and photomorphogenesis for greenhouse crops has been well investigated (Massa et al., 2008; Bugbee, 2016; Bantis et al., 2018). Until now, our knowledge of cannabis production has stemmed from experiments performed when growing cannabis was illegal (Vanhove et al., 2011). Current findings in plant photobiology and lighting control will provide the information needed by horticultural scientists to establish optimal cannabis production protocols and to maximize cannabinoid yields. To this end, this review focuses on recent developments and our current understanding of photosynthesis and photomorphogenesis in greenhouse crops, with the latest reports on cannabis production in order to adequately inform the industry on the importance of lighting control for cannabis growth and cannabinoid production. A brief overview of the cannabis profile is provided, and three main topics are explored: (1) light, photosynthesis, and photosynthetically active radiation (PAR); (2) photomorphogenesis, plant photoreceptors, and secondary plant metabolites; and (3) electrical lighting systems.

## Cannabis Profile

The cannabis plant is the one of the oldest plant sources for food, medicinal, or ritual use (Kriese et al., 2004; Chandra et al., 2017). Today, cannabis is often referred to as marijuana, a term used to describe a female cannabis plant that produces flower buds, as opposed to hemp, which is grown for several industrial applications. Throughout this review, use of the term “cannabis” will refer to the female cannabis (*C. sativa*) plant with high psychoactive properties. Cannabis plants synthesize and accumulate 60–85 different psychoactive cannabinoids in their budding structures, and these are directly associated with cannabis consumption (El-Alfy et al., 2010). The most abundantly produced cannabinoids in cannabis plants are tetrahydrocannabinol [THC; or Δ^9^-tetrahydrocannabinol (Δ^9−^THC), cannabidiol (CBD), and the primary product of THC-degradation, cannabinol (Benson et al., 1999)]. The most psychoactive cannabinoid is THC, and its pharmacology has been well studied (El-Alfy et al., 2010). Over the last few years, CBD has drawn significant attention since its reported therapeutic potential as a treatment for intractable pediatric epilepsy (Friedman and Devinsky, 2015).

The *Cannabis* genus is commonly conceived as only constituting a single species. However, *C. sativa* L. may be divided into three sub-species: *C. sativa* ssp*. sativa*, *C. sativa* ssp. *indica*, and *C. sativa* ssp*. ruderalis*. The first two species, often referred to as “Sativa” and “Indica”, are the main cannabis plant species of recreational and medicinal interest (McPartland, 2017). They have distinct yet opposing THC and CBD ratios; *C. sativa* ssp. *indica* typically possesses a high THC to CBD ratio (Fischedick et al., 2010), whereas the reverse is known for *C. sativa* ssp*. sativa.* In today’s marketplace, however, these distinctions are almost meaningless as new strains have been created from crossbreeding. *C. ruderalis* is the least known subspecies, and it is not commercially produced because of low plant yields (Fischedick et al., 2010).

## Light, Photosynthesis, and Photosynthetically Active Radiation (PAR)

Light is one of the most important environmental parameters that impacts plant growth and development. It exerts a vast range of effects on photosynthetic activity and photomorphogenic responses throughout the plant’s life (Pocock, 2015; Naznin et al., 2016; Ouzounis et al., 2016). Close to half of the sun’s total radiation emission reaching the Earth’s surface is visible light, ranging from 400 to 740 nm wavelengths (Both et al., 2015). Visible light is flanked by shorter wavelengths and invisible ultra-violet (UV) electromagnetic radiation (10–400 nm) and by infrared radiation (IR; 700–1 mm); this roughly constitutes the remaining half of the solar radiation incident on the Earth’s surface (Cooper and Hausman, 2004). These three wavelength regions of the electromagnetic spectrum are the most significant with respect to biological systems (Mishra, 2004). Visible light includes violet (~400–450 nm), blue (~450–520 nm), green (~520–560 nm), yellow (~560–600 nm), orange (~600–625 nm), red (~625–700 nm), and far-red (FR; > 700 nm). The most important part of the light spectrum for plants, PAR (400–700 nm), falls within the visible light range (McCree, 1972a,b; van Iersel, 2017).

### The Basis of Photosynthesis

Photosynthesis plays a critical role in plant growth, as there is a close correlation between plant productivity and their photosynthetic rates in a given environment (Zelitch, 1975). Photosynthesis defines the complex set of reactions by which plant and phototrophic cells harvest, transfer, and store light energy as chemical potential in the carbon bonds of carbohydrates (Cooper and Hausman, 2004). Photosynthesis occurs within the chloroplast, a chlorophyll-bearing plastid organelle dedicated to energy production (Cooper and Hausman, 2004; Mishra, 2004). Chloroplasts are mostly found in the cytoplasm of palisade and spongy mesophyll cells located between the bounding epidermal layers of leaves (Mishra, 2004). The energy-generating, photooxidation-reduction reactions of photosynthesis occur within the third, internal thylakoid membrane system of the chloroplast; it forms networks of flattened thylakoid disks, often stacked in grana (Cooper and Hausman, 2004). Embedded in the thylakoid membrane are five-membrane protein complexes that serve in electron transport and the concomitant synthesis of the energy carrier molecules NADPH and ATP, fueling carbohydrate synthesis. Prominent among these are the two main photosynthetic light reaction centers, membrane protein photosystem I and II complexes (PSI and PSII), named after the order of their discovery yet counterintuitive to their evolution in nature (Cooper and Hausman, 2004).

**Figure 1:**
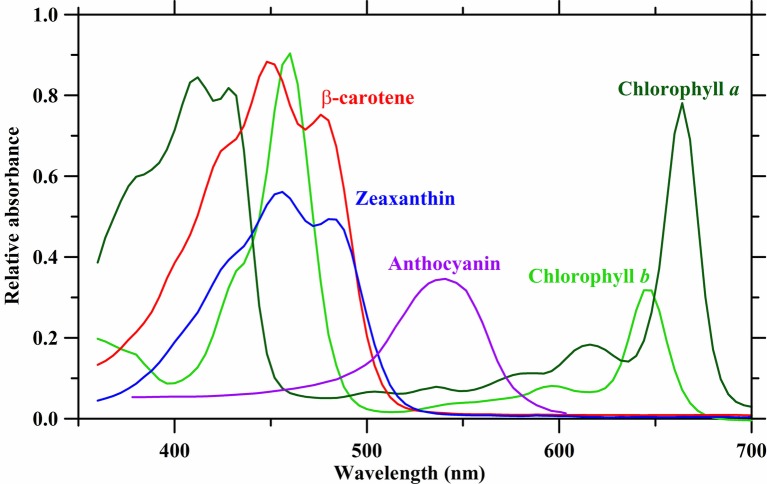
Absorbance spectra of plant photosynthetic pigments in acetone. Absorbance data are derived from Taiz and Zeiger (2002), Avital et al. (2006), Heddad et al. (2006), and Kobayashi et al. (2013).

The aforementioned photosystems contain arrays of associated chlorophyll and carotenoid antenna pigments, molecules involved in harvesting light energy for photosynthesis, organized in such a way as to maximize light energy capture and transfer. Plant pigments have specific wavelength absorbance patterns known as the absorbance spectrum (Figure 1). Chlorophylls *a* and *b* (Chl *a* and *b*) absorb wavelengths of light strongly in the red and blue regions, with less absorbance occurring in the green wavelengths. In acetone, Chl *a* exhibits peak absorbance at 430 and 663 nm, while Chl *b* peaks at 453 and 642 nm. The pigments β-carotene and lutein in acetone absorb strongly in the blue region of light with a maximum peak occurring at 454 and 448 nm, respectively (Hopkins and Hüner, 1995; Taiz and Zeiger, 2002). These pigments have local absorbance peaks, while β-carotene has a second absorbance peak at 477 nm, and lutein has two local absorbance peaks at 422 and 474 nm. However, it is important to note that peak absorbance can shift up to 38 nm and is dependent on the specific environment surrounding the chloroplasts (Heber and Shuvalov, 2005).

### Photosynthetically Active Radiation (PAR) and Standard Units for Plant Lighting

Understanding the spectral quality of photosynthesis is critical when selecting a lighting system with proper light quality and quantity for any indoor plant cultivation. Our current understanding of the spectral quality of photosynthesis is mainly based on McCree’s findings in the 1970s (McCree, 1972a). The action spectrum of plant leaves was described as the span of wavelengths from approximately 400–700 nm, over which plants absorb and effectively use radiant light energy for photosynthesis (McCree, 1972a). This brought some definition to what is now commonly known as PAR (measured in μmol m^−2^ s^−1^), the measure of that relates the intensity and rate of radiant light energy per surface area emitted by a light source from within the action spectrum of plants. To achieve this, the photosynthetic spectral quantum yield or the CO_2_ consumed by plant leaves per mole of photons absorbed was determined for 22 crop plant species by correlating the monochromatic light irradiance intensity (W m^−2^) required to obtain a certain rate of photosynthesis in leaf fragments to their absorption spectrum, measured in an integrating sphere with a spectrophotometer. The assay covered the wavelength range from 350 to 750 nm, in 25 nm waveband increments, and photosynthesis was measured based on the CO_2_ uptake rate, measured with an infrared gas analyzer based on CO_2_ differentials under dark light versus the tested wavelength band of light. Two major, distinct peaks at 440 and 620 nm were observed, followed by a secondary peak at 670 nm. To this end, McCree’s experiments first described a plant’s PAR curve, a term that defines a plant’s light action spectrum and the wavelengths used most efficiently for glucose biosynthesis and the storage of free chemical energy (McCree, 1972b; Young, 1991).

McCree (1972b) determined that quantifying PAR in quantum or photon flux units based on moles of photons would yield results that more closely correlated to the actual photosynthetic rate, since photosynthesis is a quantum photochemical process, with one carbon fixed and one molecule of oxygen evolved per roughly 10 photons (quanta) of light absorbed. Both units of measurement, radiant flux density (W m^−2^) and photon flux density (μmol m^−2^ s^−1^), are typically used to report plant lighting systems (McCree, 1972a; Inada, 1976; Both et al., 2015); however, plant yields are overestimated for blue light over red light when using radiant flux density, and this overestimation is smaller when light energy is measured in photon flux density (McCree, 1972b; Inada, 1976). Therefore, PAR is defined from 400 to 700 nm in quantum units of photosynthetic photon flux density (PPFD, μmol m^−2^ s^−1^) (McCree, 1972b; Inada, 1976; van Iersel, 2017). PPFD is broadly considered as the available estimate of potential photosynthetic flux, since the two are positively correlated. PAR is determined by integrating PPFD values within the limits of the plant action spectrum for photosynthesis (McCree, 1971, 1972b). Based on McCree’s findings on plant action spectrum, the PAR spectrum is used to integrate photon flux values, and PPFD gives an instantaneous estimate of potential photosynthetic activity with regard to measured light source emissions (Sager and Giger, 1980; Sager et al., 1982).

Although McCree (1972a,b) proved that the use of PPFD is necessary when quantifying photosynthetic productivity over four decades ago, other photometric units of light such as lumens, lux, or foot-candles are still employed. These photometric units are based on the eye’s response to brightness, where human eyes are more sensitive to green light than red or blue light. Moreover, light below 400 nm and above 700 nm induces photosynthetic activity, which was not previously considered in PAR (McCree, 1972a; Inada, 1976). This led to the use of yield photon flux. Yield photon flux weighs photosynthetic activity from 360 to 760 nm based on McCree’s quantum yield curve, under the assumption that the curve remains true with different light conditions (Sager et al., 1988; Barnes et al., 1993). Importantly, all spectral quality studies were conducted under low light intensity (< 150 μmol m^−2^ s^−1^). Whether the curve keeps its infamous form under higher light intensities or can be applied to other plants remains to be determined (Lefsrud et al., 2008). In the case of cannabis plants, most studies have been conducted under light intensities ranging from 300 to 2000 μmol m^−2^ s^−1^; this is higher than what is typically used for greenhouse crops and all spectral quality studies (McCree, 1972a; Inada, 1976; Chandra et al., 2008; Chandra et al., 2015). In this scenario, the spectral quality of photosynthesis for cannabis plants is required to optimize growth.

### Light Compensation and Saturation Points

Increased PPFD increases with plant growth and photosynthetic rate, and this linear increase occurs between the light compensation point and the light saturation point. The light compensation point is the point at which the photosynthetic activity of the plant equals its respiration activity, and the resulting CO_2_ release from respiration is equivalent to that used during photosynthesis. The light compensation point is used as a base to select an appropriate light intensity. If light intensity is below the light compensation point, there is a net loss of sugars (Noodén and Schneider, 2004). For broad spectrum light, Erwin and Gesick (2017) reported that light compensation points were 25, 13, and 73 μmol m^−2^ s^−1^ for chard, kale, and spinach, respectively.

The light saturation point is the light intensity at which the photosynthetic rate reaches its maximum, where more light has no or a negative effect on photosynthesis. Understanding the light saturation point in plants provides lighting engineers with an opportunity to provide optimal light intensities that will maximize plant growth. Light saturation points have been investigated for many greenhouse crops, including kale, spinach, and Swiss chard (Boese and Huner, 1990; Yamori et al., 2005; Dahal et al., 2012; Ruhil et al., 2015). A study using 470 and 655 nm LEDs reported that the light saturation points for kale and chard ranged between 884 and 978 μmol m^−2^ s^−1^ and at 1238 μmol m^−2^ s^−1^ for spinach (Erwin and Gesick, 2017). The light saturation point for cannabis has not yet been determined, but its net photosynthetic rates at different temperatures (25–40°C) and intensities (up to 2,000 μmol m^−2^ s^−1^) were reported (Chandra et al., 2008; Chandra et al., 2015). In these studies, no decline in photosynthesis rate was observed at the highest intensity used; however, net photosynthetic rates at 30°C decreased by ~20% from 1,500 to 2,000 μmol m^−2^ s^−1^ (Chandra et al., 2008; Chandra et al., 2015).

For any given wavelength and plant, an increase in photosynthetic rate results in increased yields until reaching the light saturation point. Therefore, additional lighting results in a similar linear increase in biomass yield that is counteracted by increased operating light-related energy costs (Terashima et al., 2009). With high-intensity LED lights, a favorable and constant light intensity above the light compensation point and below the light saturation point is required but this is species-, environment-, and grower needs-dependent (Mathieu et al., 2002; van Ieperen and Trouwborst, 2007).

## Photomorphogenesis, Plant Photoreceptors, and Secondary Plant Metabolites

Light wavelength and intensity are used to quantify light in plant lighting experiments, and it is now widely accepted that both influence photosynthesis and photomorphogenesis (Olle and Viršile, 2013; Singh et al., 2015). With the McCree curve and lighting technology improvements, photomorphogenic responses with whole plant measurements have been investigated under various wavelengths and intensities of narrow spectrum light for greenhouse crops (Hoenecke et al., 1992; Kim et al., 2004a; Li and Kubota, 2009; Stutte et al., 2009; Martineau et al., 2012). In contrast to photosynthesis that is associated with growth from direct light energy, photomorphogenesis is defined as the effect of light on plant development. Several plant responses such as germination and flowering result from the mere presence of light and are not influenced greatly by its intensity (Hall et al., 2014; Kołodziejek and Patykowski, 2015). Therefore, the outcome of a plant’s response under any light spectrum results from the interactive effects between photosynthesis and photomorphogenesis. These two responses are difficult to separate from each other for long-term whole plant growth. Note that plants grown with sunlight, whether in an outdoor environment or in a greenhouse with supplemental electrical lighting, still receive the broad spectrum of light and have corresponding photomorphogenic responses. Sunlight and electrical lighting systems are further discussed in Section Traditional Light Sources.

### Photomorphogenic Responses and Photoreceptors

**Figure 2:**
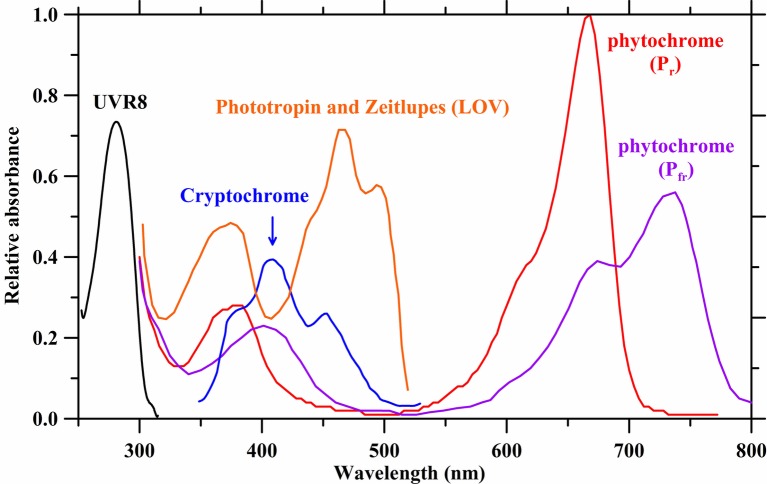
Absorbance spectra of photoreceptors. Spectrum data are derived from Taiz and Zeiger (2002), Galvão and Fankhauser (2015), and Sager et al. (1988).

Photomorphogenesis is the light-mediated development of plants regulated by five different photoreceptors (Figure 2; Folta and Carvalho, 2015; Pocock, 2015). They mediate and modulate dozens of structural plant developments such as height, leaf size, and flowering. These changes to plant architecture affect long-term plant development and subsequent photosynthetic surfaces.

### Red (~625–700 nm) and Far-Red (> 700 nm) Light

Red light impacts photomorphogenesis, leaf nutrient content, and stem growth. It is essential for chlorophyll synthesis and for straightening the epicotyl or hypocotyl hook of dicot seedlings (McNellis and Deng, 1995; Goins et al., 1997; Poudel et al., 2008; Johkan et al., 2012). These processes are under the influence of phytochrome control. Phytochrome is sensitive to red (~650–670 nm) light and far-red (FR) light (~705–740 nm), and to a lesser extent, blue light (~400–500 nm). For any one phytochrome, there exists a photoequilibrium of two interconvertible forms, red and FR absorbing forms (also known as Pr and Pfr, respectively). Pfr is the active form of phytochrome and it elicits physiological responses (Shinomura et al., 2000). Pr, the other form of phytochrome, is the inactive form that switches to Pfr upon absorbing ~650–670 nm light (Nagatani, 2010; Folta and Carvalho, 2015). In long day plants, various experiments suggest that flowering is promoted mostly when red light (or light creating a high Pfr/Pr ratio) is delivered during the early part of the photoperiod and when FR light (or light creating a lower Pfr/Pr ratio) is delivered toward the end of the photoperiod (Lane et al., 1965; Evans, 1976; Kadman-Zahavi and Ephrat, 1976; Thomas and Vince-Prue, 1996). However, certain cannabis genotypes such as “G-170” are insensitive to changes in the R:FR ratio, and no effect on flowering has been observed (Magagnini et al., 2018). The authors concluded that a low R:FR ratio during a long photoperiod (18 h light, 6 h dark/vegetative stage) is beneficial to the development of mature cuttings, contradicting popular belief in the cannabis industry.

The effect of red light on plant physiology has been investigated (Poudel et al., 2008; Vu et al., 2014). Poudel et al. (2008) reported that red light induced an increase in rooting percentage and root numbers in grape (*Vitis vinifera*) plants. Wu and Lin (2012) showed that king protea (*Protea cynaroides* L.) plantlets grown in red light produce a higher number of roots and new leaves. Vu et al. (2014) reported that “*Lapito*” tomato plants grown solely under red LED light produce a higher total root surface area, length, and number of root tips in comparison with other light treatments. Lower leaf nitrogen content was found in rice (*Oryza sativa* L.) and spinach (*Spinacia oleracea* L., cv. Megaton) grown under red light treatment (Matsuda et al., 2004; Ohashi et al., 2005; Matsuda et al., 2007). In addition, photosynthetic rate reductions observed for plants grown under red light are reportedly due to stomata being controlled more by blue light than by red light (Sharkey and Raschke, 1981; Zeiger, 1984; Bukhov et al., 1996).

Red light further regulates flowering quality, quantity, and flowering duration (Bula et al., 1991; Tennessen et al., 1994). According to Guo et al. (1998) and Thomas and Vince-Prue (1996), inhibition of flowering with red light is effected by red light receptors including phytochromes (Kelly and Lagarias, 1985). The number of visible flower buds in marigold plants was approximately five times higher when grown with fluorescent light supplemented with red LEDs, as well as under fluorescent light, when compared to monochromatic blue or red light. No flower buds formed in salvia plants when grown under monochromic blue or red light or when fluorescent light supplemented with FR light was used for marigold (*Tagetes minuta*) plants.

Plants grown under canopy shade conditions or in the proximity of other plants show a range of responses to changes in R:FR ratios of ambient light. This response, known as shade avoidance or the near neighbor detection response, is characterized by an acceleration of flowering time (i.e., becoming visible within the expanded floral bud) and rapid elongation of stems and leaves (Halliday et al., 1994; Smith, 1994). Kasperbauer (1988) determined that FR light reflected from neighboring seedlings increased the R:FR ratio plants received, inducing a density-dependent increase in stem length, chloroplast content, chlorophyll *a/b* ratio, and CO_2_ fixation rate, along with decreased leaf thickness. In recent years, the effect of FR light (or a low R:FR ratio) has been intensively investigated in different plant species and development stages (Li and Kubota, 2009; Finlayson et al., 2010; Mickens et al., 2018; Park and Runkle, 2018). Supplemental FR treatments increased dry mass for many greenhouse crops during vegetative development (Hogewoning et al., 2012; Lee et al., 2016; Mickens et al., 2018; Park and Runkle, 2018), but conflicting results on leaf area were reported. Hogewoning et al. (2012) reported no significant difference in leaf area for tomato (*L. esculentum* “Mecano”) and cucumber (*Cucumis sativus* “Venice”), whereas an increase in leaf area was observed for lettuce, petunia (*Petunia × hybrida),* geranium (*Pelargonium × hortorum*), and coleus (*Solenostemon scutellariodes*) (Lee et al., 2016; Mickens et al., 2018; Park and Runkle, 2018). Such differences in leaf area responses among species are still unknown and need to be addressed. For an extensive examination of FR light, the reader is referred to a recent review (Demotes-Mainard et al., 2016).

### Blue (~450–520 nm) and UV (< 400 nm) Light

Blue and UV-A light triggers cryptochrome (320**–**500 nm) and phototropin (phot1 and pho2; 320–500 nm) function (Jones, 2018). These two photoreceptors regulate various physiological and developmental processes including chloroplast relocation, germination, elongation, and stomatal opening, which impacts water transpiration and CO_2_ exchange (Cosgrove, 1981; Schwartz and Zeiger, 1984). Blue light mediates chlorophyll and chloroplast development, enzyme synthesis, and plant density, and regulates responses to biotic environmental stresses (Goins et al., 1997; Schuerger et al., 1997). Walters and Horton (1995) reported that blue light deficiency can impact the light saturation rate of photosynthesis and can change the Chl *a/b* ratio in *Arabidopsis thaliana*. Blue light causes thickness of the epidermis and palisade mesophyll cells in *Betula pendula* (Sæbø et al., 1995). Lee et al. (2014) concluded that shorter blue wavelengths (<445 nm) promote stem growth, plant height, and anthocyanin synthesis in green perilla (*Perilla frutescens* var. *japonica* Hara cv. *Soim*) plants. Cannabis plants grown under blue light with a short photoperiod (12 h light:12 h dark/flowering stage) improved cannabinoid content (Magagnini et al., 2018). This same study suggested that there is a synergy between UV-A and blue wavelengths that induces cannabigerol accumulation in cannabis flowers.

Blue light activates Zeitlupe (ZTL) family function, a group of proteins that plays a role in circadian clock regulation, wherein their light-dependent function allows modulation of internal timing signals (Kim et al., 2007). Accordingly, optimal lighting regimes for cannabis growth and production should take advantage of this temporal regulation initiated by the circadian clock and light-sensitive ZTL protein function.

Wavelengths of light that are shorter than the PAR spectrum [e.g., violet light and UV (<400 nm) radiation] have limited photosynthesis; however, discrete photomorphogenic effects are observed when UV-B (290**–**320 nm) sensing systems are triggered (Frohnmeyer and Staiger, 2003; Folta and Carvalho, 2015). UV-B radiation is perceived *via* the UV-B photoreceptor UV resistance locus 8 (UVR8). Although UV-B represents a threat to plant integrity in large quantities, smaller quantities of UV-B have important benefits such as promoting pest resistance, increasing flavonoid accumulation, improving photosynthetic efficiency, and serving as an indicator of direct sunlight and sunflecks (Ballaré et al., 2012; Wargent and Jordan, 2013; Zoratti et al., 2014; Moriconi et al., 2018). Further to this, some UV-B responses can also be modulated by a UVR8-independent signal and UV-A radiation, since plants’ responses to UV-B light are regulated by both UVR8-dependent and -independent pathways (Morales et al., 2013; Li et al., 2015; Jenkins, 2017). UV-B light reportedly elicits THC accumulation in both leaves and buds (Pate, 1983; Lydon et al., 1987; Potter and Duncombe, 2012).

### Green (~520–560 nm) Light

Green light is often considered unavailable for plant growth since plant photosynthetic pigments have limited absorbance for these wavelengths. However, there is evidence that green light is available for active plant growth, yet this phenomenon is wavelength- and intensity-dependent (Kim et al., 2004a; Kim et al., 2005; Johkan et al., 2012). Green light influences plant morphology, including leaf growth, stomatal conductance, and early stem elongation (Folta, 2004; Kim et al., 2004a,b). Kim et al. (2004) first examined the effect of green light on plant growth and photomorphogenesis, later concluding that it impacted plant growth at low light intensity (~150 μmol·m^−2^·sec^−1^) (Kim et al., 2005). A low percentage (≤ 24%) of green light enhanced plant growth, whereas plant growth was inhibited under a higher percentage of green light (Kim et al., 2004a, 2005; Folta and Maruhnich, 2007; Lee et al., 2011; Liu et al., 2017). Lee et al. (2011) reported that lady’s slipper orchid grown under a combined LED lighting regime (8:1:1 ratio; 660 nm, 525 nm, and 450 nm) had at least 60% greater shoot dry mass when compared to blue or red LED emissions alone, or to a combination of red and blue lights at the same light intensity. Furthermore, green light exhibits better leaf tissue penetration ability (Brodersen and Vogelmann, 2010), resulting in better plant canopy penetration than either red or blue light (Klein, 1992). The issue with green light is that it exerts an antagonistic effect on other blue light-induced responses, including stomatal closure (Frechilla et al., 2000) or anthocyanin accumulation (Zhang and Folta, 2012). In cannabis plants, THC levels are negatively affected by the presence of green light (Mahlberg and Hemphill, 1983; Magagnini et al., 2018).

### Secondary Plant Metabolites

Secondary plant metabolites such as carotenoids, flavonoids, and anthocyanins accumulate in plant cells and leaves as light-screening compounds to limit damage caused by high light intensity and UV radiation (Takahashi and Badger, 2011; Darko et al., 2014).

#### Carotenoids

Carotenoids are photosynthetic accessory pigments that have absorbance spectra in the 400–550 nm region (Frank and Cogdell, 1996). Carotenoids prevent photo-oxidative damage caused by the photosynthetic light harvesting apparatus and other cell components by thermally dissipating the excess energy of the single excited chlorophyll (^1^Chl*) and possibly a triplet excited chlorophyll (^3^Chl*) within light reaction centers, as well as scavenging any evolved singlet-oxygen (^1^O_2_) (Müller et al., 2001; Mozzo et al., 2008).

#### Terpenes

Although present in much smaller quantities than cannabinoids, most terpenes in cannabis plants (e.g., monoterpenes and sesquiterpenes) are located in the glandular trichomes and are functionally diverse (Malingre et al., 1975; Turner et al., 1980). Terpenes are volatile aromatics that impact or contribute to the taste and smell of plants (Goff and Klee, 2006), defend against biotic stresses (Martin et al., 2003), and are plant hormones that regulate growth (Milborrow, 2001; Sakakibara, 2005; Hedden and Thomas, 2012). In addition, some terpenes help plants manage light and drought stress (Buchanan et al., 2000). Studies have demonstrated a relationship between terpene biosynthesis and light (Loveys and Wareing, 1971; Gleizes et al., 1980; Yamaura et al., 1991). Schnarrenberger and Mohr (1970) and Tanaka et al. (1989) both observed that carotenoid and monoterpene biosynthesis is regulated by the red light photoreceptor, phytochrome.

#### Cannabinoids

Cannabinoids are synthesized in secretory cells inside glandular trichomes, which are highly concentrated in unfertilized female flowers before senescence (Potter, 2004, 2009). Shoyama et al. (2008) found that cell death was induced when cannabis leaves secrete cannabinoids from glandular trichomes into leaf tissue. Lydon et al. (1987) reported increased THC concentrations when cannabis plants were grown with supplemental UV-B radiation, suggesting that cannabinoids may play some role in UV protection. Limited published research exists on the role of cannabinoids in cannabis plants.

#### Flavonoids

Flavonoids are sensitive to light quality, and flavonoid concentrations in plants are higher when grown under UV, blue, and FR light treatment (Fu et al., 2016; Pedroso et al., 2017; Liu et al., 2018). The two-ring, 15-carbon, general structure of flavonoids makes this group structurally and functionally diverse. Flavonoids comprise many classes (flavonols, flavones, flavanones, anthocyanins, and isoflavonoids) that are defined by various accessory groups attached to the central 15-carbon skeleton (Iwashina, 2000). This allows for their important roles as pollinator and feeding attractants, oviposition stimulants, and feeding deterrents, as well as in plant disease resistance and managing light stress (Hamamura et al., 1962; Ingham, 1972; Arakawa et al., 1985; Noh and Spalding, 1998; Nishida, 2005; Goff and Klee, 2006). Optimal lighting systems for cannabis growth and production must include an optimal light spectrum for flavonoid production. UV, blue, and FR are beneficial wavelengths that should be given greater consideration.

## Electrical Lighting Systems

Electrical lighting systems usually serve as supplemental lighting for photoperiod control, to increase light intensity in a greenhouse, or as sole lighting for indoor plant production. Electrical lighting systems available for plant growth include incandescent bulbs, fluorescent bulbs, high pressure sodium (HPS) lamps, and LEDs. All of these light sources have been used throughout the history of cannabis production (Potter, 2009). For instance, fluorescent bulbs and HPS lamps are mainly used for young cuttings and during the flowering stage, respectively. For the vegetative growth stage, a wide variety of lighting types have been reported; these include metal halide bulbs, HPS lamps, LEDs, or a combination of different lighting types (Sweet, 2016; Chandra et al., 2017).

### Traditional Light Sources

**Figure 3:**
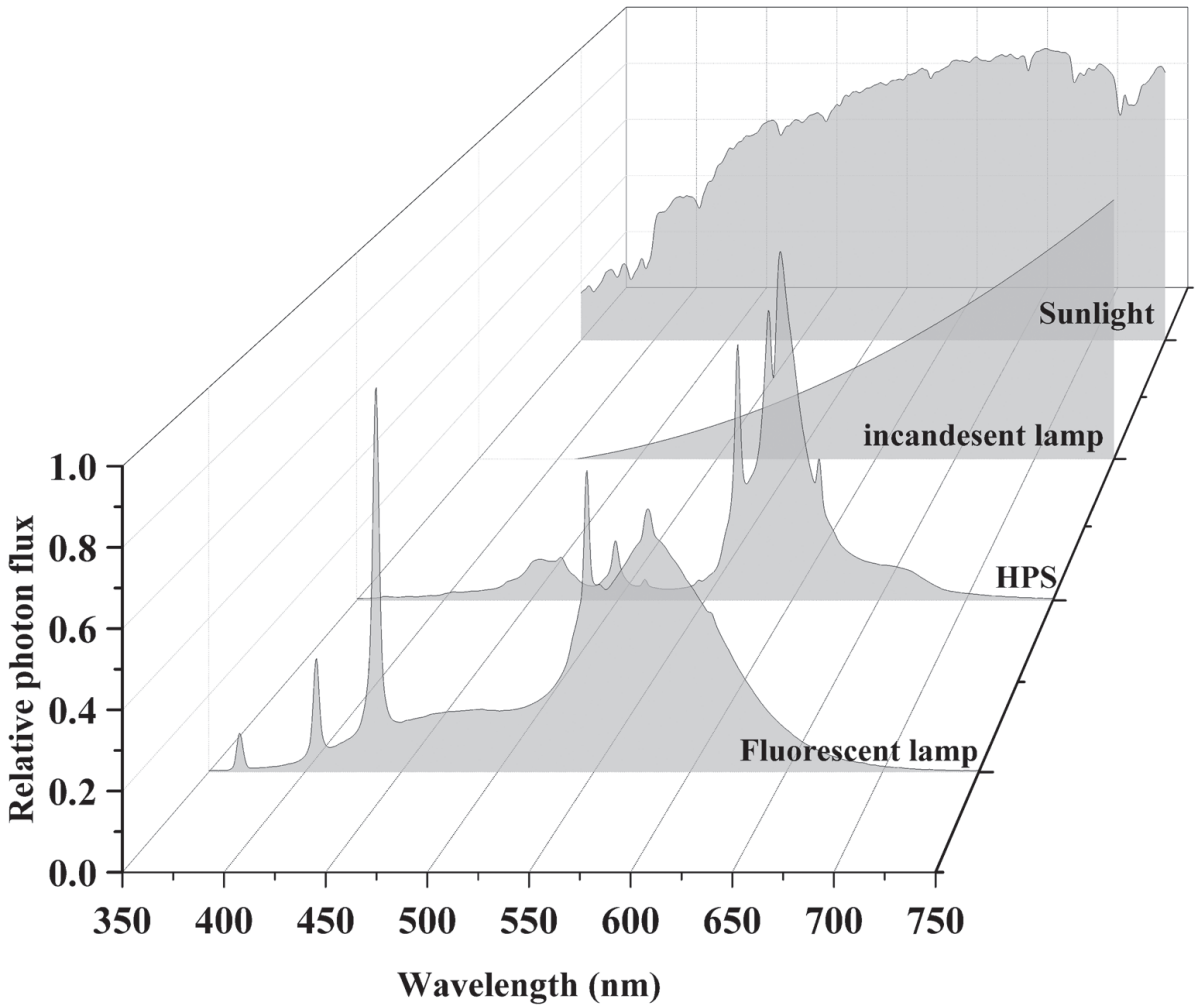
Sunlight and traditional light source spectra. Data were collected using a spectroradiometer (PS-300, Apogee, UT).

Sunlight and traditional light source spectra are shown in Figure 3. Incandescent light bulbs are composed of an airtight glass bulb and a tungsten filament that emanates electromagnetic radiation in the visible spectrum upon being heated (Kitsinelis, 2016). Visible light is emitted as the filament reaches ~2,800 K, with intensity increasing from 400 to 700 nm (Gupta and Agarwal, 2017). Most energy is emitted as FR light and only 60% of light energy is within the PAR spectrum. Its luminous efficiency never exceeds 20 lumens per watt (lm/W), and the energy conversion efficiency ranges from 1 to 5% (Gupta and Agarwal, 2017). The low luminous efficiency of incandescent light compared to other lighting systems has led to the phasing out of incandescent light bulbs, and they have limited applications for cannabis cultivation.

Gas discharge lamps include fluorescent bulbs, high-intensity discharge lamps, and metal halide lamps. Fluorescent bulbs are low-pressure mercury vapor discharge lamps that produce UV light *via* the ionization of the gaseous metal ions, which excite a phosphor coating that results in a visible light fluorescence. The energy conversion efficiency of fluorescent bulbs are below 30% (Shur and Zukauskas, 2005), yet the spectral quality of fluorescent bulbs has 90% of its emitted photons in the PAR spectrum (Gupta and Agarwal, 2017). The lifespan of fluorescent lamps, however, depends on starting and stopping frequencies since the emissive coating (usually phosphor) on the electrodes slowly evaporates during operation and rapidly erodes during start-up. Fluorescent bulbs are usually used for the establishment of seedlings or young cuttings of cannabis plants with an 18-h photoperiod before transplanting (Chandra et al., 2017).

High-intensity discharge lamps operate under the same working principles as fluorescent bulbs, apart from being operated at high pressures and temperatures. High-intensity discharge lamps are classified into three types based on the vapors used: sodium, mercury, and metal halide. High-pressure mercury lamps have a luminous efficiency of 60 lm/W, whereas HPS lamps have a luminous efficiency between 80 and 125 lm/W. HPS lights not only emit most strongly in the yellow light (560–600 nm) of the PAR spectrum but also emit IR that is not useful for photosynthesis (Gupta and Agarwal, 2017). In both general horticultural and cannabis production industries, HPS lamps are widely used but have disadvantages. Firstly, high heat outputs (>200°C) dramatically increase temperatures in the propagation room without proper thermal management. Secondly, although HPS lamps are rated for a longer lifespan (24,000 h) compared to fluorescent lamps, frequent starts will reduce the lifespan of HPS lamps, as does excessive lamp voltage (power surges). Metal halide lamps are modified high-pressure mercury vapor lamps. Spectral quality and intensity are controlled and have more visible wavelengths with the use of metal halides and mercury vapor. In addition, the spectral quality of the emitted radiation can be manipulated with the use of different metals and inert gases, producing light with a high luminous efficiency from 100 to 120 lm/W (Gupta and Agarwal, 2017).

### Light Emitting Diodes

**Figure 4:**
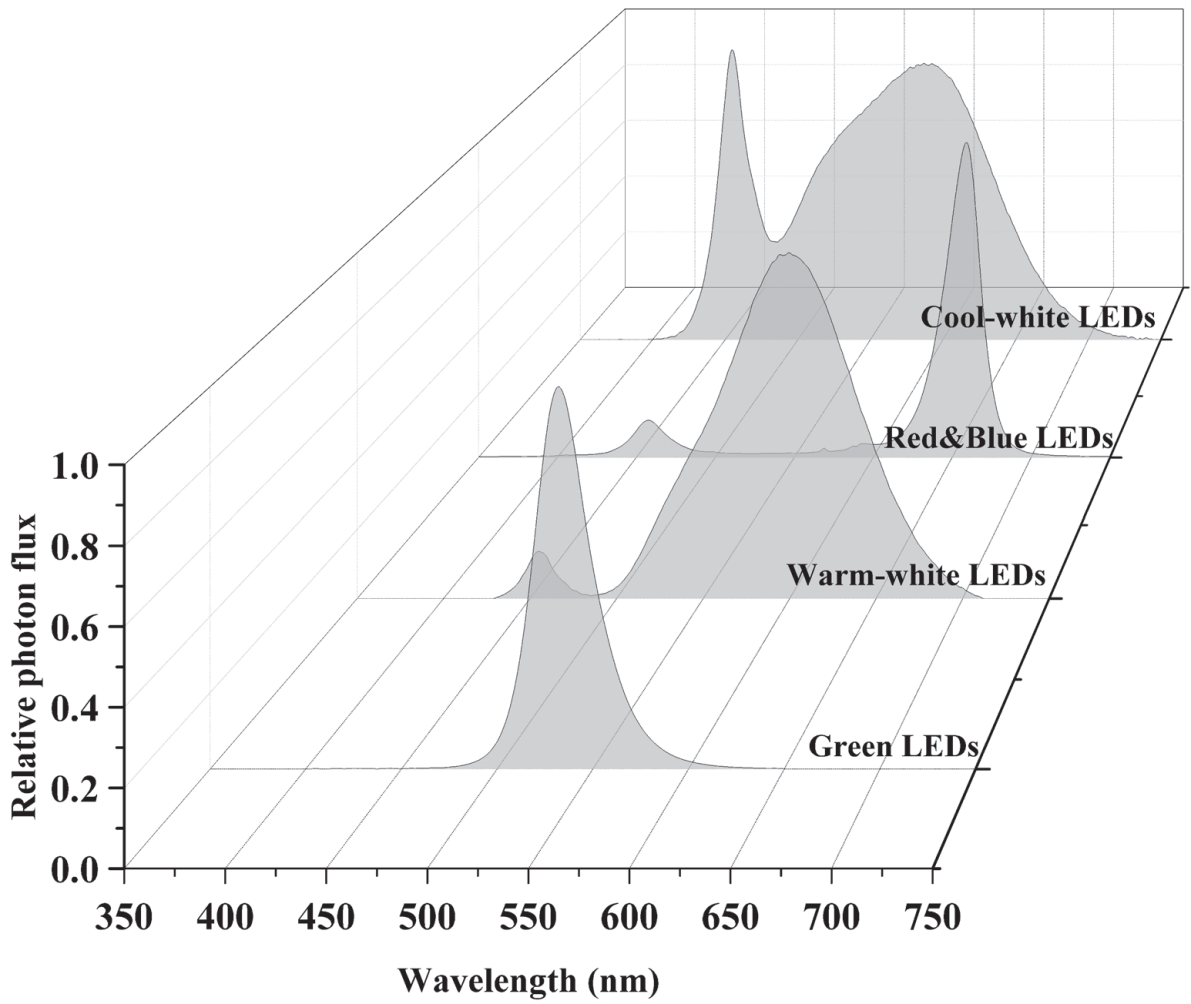
Different LED light spectra. Data were collected using a spectroradiometer (PS-300, Apogee, UT).

LEDs are an emerging, versatile artificial light source offering many advantages over other conventional artificial light sources. Advantages include high photoelectric conversion efficiency (~50%), long lifespan (30,000–50,000 h), narrow spectral emissions (~10 nm), and adjustable light intensity and quality to investigate the effects of many different spectral combinations of wavelengths on plant growth and development (Chang et al., 2012; Olle and Viršile, 2013). LED working principles and history have been extensively reviewed elsewhere (Morrow, 2008; Yeh and Chung, 2009; Singh et al., 2015; Cho et al., 2017; De Cesari et al., 2017; Viršilė et al., 2017) and will not be repeated in this review. Typical LED spectra used in the general horticultural industry are shown in Figure 4.

Apart from versatility, LEDs can address the challenge of low light intensity within the plant canopy (Massa et al., 2005). In HPS and overhead LED lighting systems, the top of the canopy is often light saturated, while the whole canopy remains light-limited. Providing additional light to the lower canopy increases the proportion of light used for photosynthesis without exceeding the point of photosynthetic light saturation (Massa et al., 2005). Unlike HPS that dissipate heat toward the illuminated plane, LEDs dissipate their heat away from its illumination plane, thereby emitting little heat (Nelson and Bugbee, 2014). Producing significantly lower leaf temperatures, they can be used for close-canopy applications, making them a practical interlighting system in commercial settings. For example, a cowpea (*Vigna unguicultata* L. Walp.) canopy irradiated by intra-canopy LEDs improved biomass production, whereas plants grown under overhead lights produced less biomass and had a reduced energy conversion rate than plants grown with intra-canopy lights. When quantified, overhead-lighted plants averaged 75% the productivity of intracanopy-lighted plants (Massa et al., 2005).

### Spectral Effects on Cannabis Production

Cannabis yield data often refers to dried floral material and corresponding cannabinoid content (Vanhove et al., 2011; Potter and Duncombe, 2012; Chandra et al., 2015). Dried bud yield may be presented on the basis of mass per plant (g per plant) or mass per unit growing area (g m^−2^) (Table 1; Rosenthal, 2010; Potter and Duncombe, 2012; Vanhove et al., 2012). Currently, there is no “standard” unit to represent dried bud yield data. In recent years, unit mass per wattage of electrical energy consumed by the lighting system (g W^−1^), has been used, since it reflects the correlation between light intensity, cannabis growth, and lighting system efficacy (Hough, 2003; Potter and Duncombe, 2012). Depending on the cannabis plant variety, yield data in g W^−1^ varies between 0.9–1.6 g W^−1^, and some growers claim that the “standard” unit is 1 g W^−1^ (Potter and Duncombe, 2012).

**Table 1:** A comparison of cannabis yield data compiled from published reports (Vanhove et al., 2011; Potter and Duncombe, 2012; Vanhove et al., 2012; Caplan et al., 2017; Magagnini et al., 2018).

Source	Light source	Strain	Dried floral yield		THC (%)	CBD (%)
			g plant^−1^	g m^−2^		
Vanhove et al. (2011)^1^	HPS (600 W)	Big Bud	9.91	142.51	15.30	0.30
		NLX	11.63	186.15	10.90	0.20
		Super Skunk	18.58	338.54	14.30	0.30
		White Widow	8.91	142.52	9.70	0.20
Vanhove et al. (2012) ^2^	HPS (600 W)	Big Bud	48.14	577.69	–	–
		Skunk #1	52.11	625.35	–	–
		Silver Haze #9	61.96	743.47	–	–
		X	45.78	549.33	–	–
Potter and Duncombe (2012) ^3^	HPS (600 W)	–	–	544	14.49	–
Caplan et al. (2017)	Fluorescent light	OG Kush Grizzly	41.6	270.40	10.60	0.08
Magagnini et al. (2018)	HPS	G-170	26.2	–	9.50	0.10
	RB LED		23.1	–	13–15	0.15
	RGB LED		22.8	–	15.40	0.20

1 Reported plant density of 16 m^−2^.

2 Reported plant density of 12 m^−2^.

3 Mean values for seven strains.

Cannabis plants have been cultivated under different lighting systems (Lydon et al., 1987; Chandra et al., 2008, 2015; Potter, 2009; Potter and Duncombe, 2012; Hawley, 2018; Magagnini et al., 2018). Lydon et al. (1987) and Marti et al. (2014) studied the effect of UV radiation on cannabis growth and cannabinoid profiles. Lydon et al. (1987) reported that supplementing with UV-B radiation for 3 h daily increased THC concentrations on *C. sativa* leaves and buds, whereas supplementing with UV-C radiation (100–280 nm) influenced resveratrol and piceid levels (Marti et al., 2014). Photosynthetic responses in *C. sativa* were measured at different light intensities, temperatures, and CO_2_ concentrations (Chandra et al., 2008, 2011a, 2015). Of the environmental conditions tested, the highest net photosynthetic rates occurred at 30°C and 1,500 μmol m^−2^ s^−1^, but this was reduced by nearly 20% when intensity increased to 2000 μmol m^−2^ s^−1^; no declined trend was observed at any other test temperatures (Chandra et al., 2008). At 25°C, an increase in net photosynthetic rates with intensity was observed (Chandra et al., 2015). Further, elevated CO_2_ concentrations resulted in increased photosynthetic activity but had variety-specific responses (Chandra et al., 2011a).

Studies have reported that light spectrum influences cannabinoid quality and cannabinoid secondary metabolite production (Hawley, 2018; Magagnini et al., 2018). Magagnini et al. (2018) compared overhead HPS lamps to LEDs with two different light spectra (peaks at ~450 and 620 nm, as well as at ~450, 550, and 660 nm). THC percentages in *C. sativa L.* flowers were 9.5 and 15.4% for LEDs and HPS, respectively, at 450 μmol m^−2^ s^−1^. Other cannabinoids such as CBD and cannabigerol showed higher concentrations under LED light treatments compared to HPS light. Hawley (2018) reported that combining 530-nm LED light, 440-nm LED light, 655-nm LED light, and metal halide lamps increased dry bud yield by 18–24% relative to the control. The same trends were observed with cannabinoid and terpene concentrations (Hawley, 2018). This up-regulation of secondary metabolites resulted in the up-regulation of IPP and DMAPP; both are precursors for terpenes and cannabinoids. In addition to environmental factors, studies reported that strain and plant density should be considered when estimating cannabis yield (Toonen et al., 2006; Vanhove et al., 2011; Potter and Duncombe, 2012; Vanhove et al., 2012).

Although beyond the scope of this review, it is still worth mentioning the importance of other environment conditions such as temperature, relative humidity, air circulation, fertilizer rate, substrate, pH, and electrical conductivity (EC), all of which are critical for optimal cannabis growth. For cannabis plants, the ideal temperature is between 25 and 30°C, yet this may vary depending on the genetic makeup and growth behavior of each plant strain (Chandra et al., 2008, 2011b). Recommended relative humidity levels are 75% during the development stage and 55–60% during the vegetative and flowering stages (Chandra et al., 2017); however, humidity as high as 90% has been reported for the propagation stage (Hawley, 2018; Magagnini et al., 2018). In the growing room, constant airflow and drier air are also recommended to prevent plant diseases and mold formation (Chandra et al., 2017). An optimized fertilizer rate of 351 mg nitrogen per liter (N/L) for cannabis was achieved by supplying a range of nitrogen concentrations (117–585 mg N/L) in a coir-based substrate with EC ranging between 0.9 and 3.9 mS·cm^−1^ and pH ranging between 6.74 and 7.16 (Caplan et al., 2017). A growing number of studies reporting optimal values for each of the aforementioned conditions for cannabis growth indicate that they have not yet been fully elucidated, particularly with respect to the individual cultivars.

### LEDs Versus HPS Lamps

The ideal lighting system for cannabis growth is difficult to determine as both LEDs and HPS each have their respective advantages (Viršilė et al., 2017). For large scale of production with uniformly spaced plants, HPS provides a broader uniform light distribution that can cover a larger area of production than LEDs (Nelson and Bugbee, 2014). However, LEDs can be optimized to specific production conditions by controlling periodicity, quantity, and spectrum of the light provided (Pinho et al., 2007). LEDs allow high-density production systems to have a focused spectral quality that can maximize radiation transfer to plants (Nelson and Bugbee, 2014). Their low heat emission allows them to be placed in the plant canopy for maximum cannabinoid yields (Viršilė et al., 2017; Hawley, 2018).

Based on the cost analysis, photon efficacy, and capital costs of fixtures per photon delivered, it has been determined that LED fixtures cost five to ten times more than HPS fixtures, and that current, efficient fixtures available in the US have nearly identical efficiencies of 1.66–1.70 μmol J^−1^ (Nelson and Bugbee, 2014). The same study showed that both technologies have relatively low long-term maintenance costs. Dutch and Danish LED fixtures with efficiencies of 2.2–2.4 μmol J^−1^ are available in Europe, whereas the newest HPS lamps (1,000 W) reach up to 2.1 μmol J^−1^, indicating that LEDs are fully implementable on a commercial scale (Ouzounis et al., 2015).

## Summary and Future Perspectives

This review provides an outline of the impact of light on cannabis growth. Drawing on previous plant studies of other horticultural crops and using existing research performed on the cannabis plant, plant responses to different irradiance, wavelength, and photoperiods are summarized. The existing literature has demonstrated that both HPS and LEDs present viable lighting system options with possible benefits, but knowledge gaps remain with respect to cannabis production. To bridge these gaps, we propose several areas of focus for future experiments: (1) determine the effect of spectral quality on cannabis plant growth, particularly under high light intensities, as our current knowledge of spectral quality is based on typical greenhouse crops at moderate temperature (20–25°C) and it is not yet known if we can apply the McCree PAR curve to cannabis plants; (2) determine the effect of environmental conditions such as temperature and humidity on different cannabis development stages, as current recommendations are ambiguous and mostly refer to vegetative and flowering stages; (3) determine the effect of light wavelength and intensity on photomorphogenesis (for each development stage) and final cannabis yield; (4) determine the effect of microclimate and different lighting systems on cannabis plant yield. For instance, investigating the effect of sole electrical lighting systems on indoor cannabis growth, and studying how airflow, temperature, and carbon dioxide might impact whole plant growth in these microclimates; (5) determine the effect of light on nutrient uptake in cannabis while examining substrate interactions and nutrient availability across different EC and pH ranges. In all, applied research will provide proven and reliable information that may ease cannabis plant production in this fast-paced and growing industry.

## Author Contributions

SB led the writing of this manuscript, edited it, and proofread it. B-SW contributed to writing the manuscript and provided the four figures. A-SR contributed to writing and was an active editor. SM contributed to the paper and was the major editor. ML contributed to writing the manuscript and is the correspondence author.

### Conflict of Interest Statement

The authors declare that the research was conducted in the absence of any commercial or financial relationships that could be construed as a potential conflict of interest.

## Abbreviations

CBD: cannabidiol
FR: far red
HPS: high pressure sodium
IR: infrared radiation
LED: light emitting diode
PAR: photosynthetically active radiation
PCET: proton-coupled electron transfer
PPFD: photosynthetic photon flux density
THC (or Δ9: THC): tetrahydrocannabinol (Δ^9^-tetrahydrocannabinol)
UV: ultraviolet.
